# Discovering relations between indirectly connected biomedical concepts

**DOI:** 10.1186/s13326-015-0021-5

**Published:** 2015-07-06

**Authors:** Dirk Weissenborn, Michael Schroeder, George Tsatsaronis

**Affiliations:** DFKI Projektbüro Berlin, Alt-Moabit 91c, Berlin, 10559 Germany; Biotechnology Center, Technische Universität Dresden, Tatzberg 47/49, Dresden, 01307 Germany

**Keywords:** Relation discovery, Biomedical concepts, Text mining

## Abstract

**Background:**

The complexity and scale of the knowledge in the biomedical domain has motivated research work towards mining heterogeneous data from both structured and unstructured knowledge bases. Towards this direction, it is necessary to combine facts in order to formulate hypotheses or draw conclusions about the domain concepts. This work addresses this problem by using indirect knowledge connecting two concepts in a knowledge graph to discover hidden relations between them. The graph represents concepts as vertices and relations as edges, stemming from structured (ontologies) and unstructured (textual) data. In this graph, path patterns, i.e. sequences of relations, are mined using distant supervision that potentially characterize a biomedical relation.

**Results:**

It is possible to identify characteristic path patterns of biomedical relations from this representation using machine learning. For experimental evaluation two frequent biomedical relations, namely *“has target”*, and *“may treat”*, are chosen. Results suggest that relation discovery using indirect knowledge is possible, with an *AUC* that can reach up to 0.8, a result which is a great improvement compared to the random classification, and which shows that good predictions can be prioritized by following the suggested approach.

**Conclusions:**

Analysis of the results indicates that the models can successfully learn expressive path patterns for the examined relations. Furthermore, this work demonstrates that the constructed graph allows for the easy integration of heterogeneous information and discovery of indirect connections between biomedical concepts.

**Electronic supplementary material:**

The online version of this article (doi:10.1186/s13326-015-0021-5) contains supplementary material, which is available to authorized users.

## Background

### Motivation and objectives

Knowledge discovery is an important field of research, especially in the biomedical domain, in which the scale and growth of accumulated knowledge of all kinds is already beyond the capabilities of a single human to keep up with. This has motivated research towards mining knowledge from heterogeneous data of both structured and unstructured knowledge bases (KBs). The parallel use of structured and unstructured data is important because they are complementary. Structured KBs contain explicit but inadequately covered knowledge. In contrast, unstructured KBs contain nearly all of the domain specific knowledge but lack in simplicity with regards to automated analysis.

**Figure 1:**
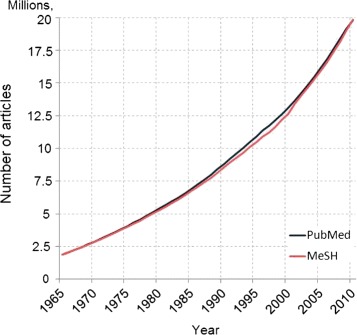
Growth of PubMed indexed scientific literature since 1965. The figure plots the number of PubMed indexed articles per year, for the period 1965-2010. The plot shows that the indexed literature grows exponentially (blue line). In parallel, the annotation of the PubMed articles with MeSH terms has so far managed to follow this growth (red line)^a^.

**Figure 2:**
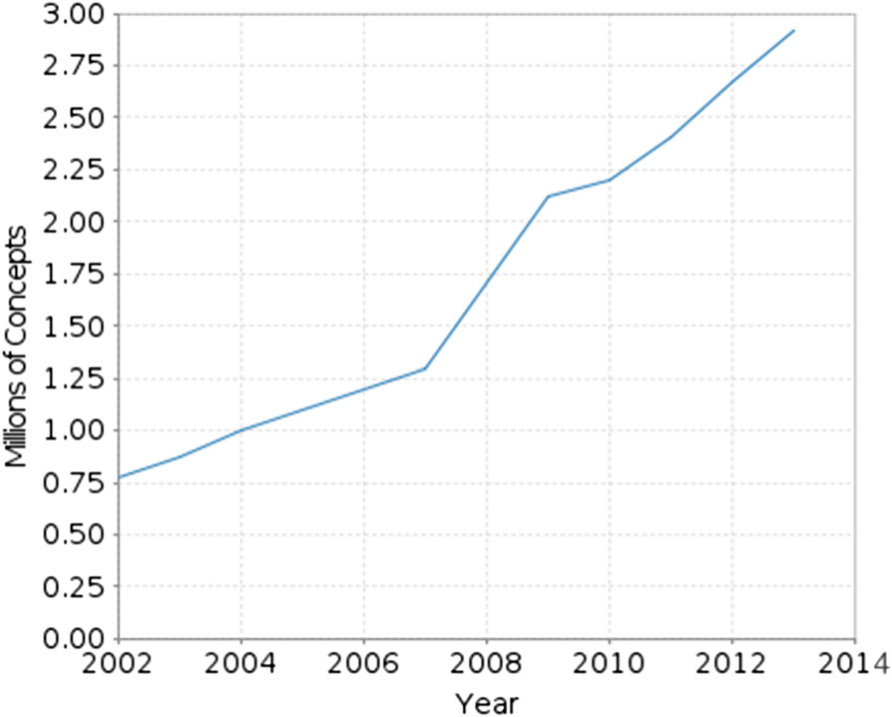
Growth of UMLS Metathesaurus in the past decade. In this plot, the growth of the UMLS metathesaurus in terms of number of included concepts is presented. The plot refers to the period from 2002 until the present. For this past period, the growth curve is steep, approximating an exponential tendency^b^.

An example of how fast the reporting of scientific findings grows in this domain is illustrated in Figure 1, where the number of scientific publications indexed by PubMed is shown to be increasing in an exponential fashion over the past decades. Similar findings can be observed for structured data by examining the growth of a representative database in the biomedical domain, namely the *Unified Medical Language System* (UMLS), shown in Figure 2.

Besides the obstacles that the large scale of the data brings into the task of extracting information there is also the issue of combining pieces of knowledge together to cover as many aspects as possible which can potentially lead to new knowledge. For example, typical information extraction techniques focusing on drugs aim at extracting targets, adverse effects and indications, which cannot succeed by limiting the applied methods to a small fragment of drug related information. Hence, it is necessary to combine facts in order to formulate hypotheses or draw conclusions about the domain concepts. This work attempts to address this problem by using indirect knowledge connecting two concepts to discover hidden relations between them.

In contrast to relation extraction, which aims at recognizing direct mentions of relations within a sentence or document between two concepts, relation discovery between indirectly connected concepts attempts to find hidden, yet unknown relations that can be derived from sequences of already known and established facts. The first reported and most famous discovery of this kind was the finding of Swanson in 1986 that fish oil may treat Raynaud’s syndrome [1]. He came to this conclusion by combining the two simple facts from different scientific studies that on the one hand fish oil has beneficial effects on blood viscosity and on the other hand patients suffering from Raynaud’s syndrom demonstrate increased blood viscosity. Until that time there was no direct connection between the concepts of fish oil and Raynaud’s syndrome, but only an indirect connection through the concept of blood viscosity, which indicated that fish oil may treat Raynaud’s syndrome which was indeed verified in 1989.

To be able to extract indirect connections between concepts, knowledge from all sources is represented by a graph comprising concepts as vertices and labelled edges connecting the concepts. Edges are created by either extracting explicit knowledge from structured databases in form of triples or by analysing unstructured textual data. The idea of using graphs to represent knowledge to find connections between concepts is not novel. It has been exploited in both ontology based [2] and literature based approaches [3]. Representing knowledge in such a way provides a simple framework that is potentially easy to interpret, makes the integration of heterogeneous data straightforward and is useful for finding indirect connections, i.e. paths, in the graph between concepts.

The task of finding connections between two concepts and identifying their meaning is called *relation discovery*. Besides being able to recognize that some connection exists it is also important to understand what kind of relation is expressed to discover the hidden relations represented by the given connections.

Relation discovery is performed on top of the aforementioned graph representation by using supervised machine learning to learn path patterns that frequently occur between concept pairs of a specific relation and can therefore be considered characteristic for that relation. A trained model can in turn be used to discover a specific relation between indirectly connected concept pairs.

This work extends the study for DILS 2014 by the introduction of a new approach and the validation on a manually created drug repositioning dataset. Furthermore, the approach is explained and discussed in great detail with additional inspections into the clustering of relations by LDA. Our main contributions lie in the joint exploitation of linguistic information and structured knowledge in a simple, extensible graph representation for fully automatized, indirect discovery of relations.

### Related work

Most work on knowledge discovery from unstructured, textual data focuses on extracting relations between two concepts mentioned in one sentence. This is very important for many applications such as the curation of databases. However, in his famous work Swanson has shown the potential of combining facts from different sources to discover new, yet unknown knowledge [1].

Recently, many studies have been conducted on finding hidden relations between concepts indirectly. Most of these works are purely based on statistical analysis of concept co-occurrence profiles from MEDLINE, which differs from our approach in that they do not take any linguistic information into account, e.g., Frijters et al. [4] and Cohen et al. [5].

Srinivasan P. et al. [6] developed a system that discovers relations by searching for interesting paths between two concepts from a start concept of interest through a set of co-occurring concepts of predefined types from MEDLINE that in turn co-occur with a set of potential target concepts of predefined types without exploiting existing linguistic information. The main difference is that the whole process is manually guided and intended to aid scientists in the search of new relations whereas our approach is completely automated. Furthermore, no machine learning is applied to find interesting connections, but only a hand-made weighting scheme based on the ideas of TF-IDF. More sophisticated studies building upon this idea include the work of Hristovski et al. [7] and Vidal et al. [8].

BioLiterate, a system developed by Goertzel et al. [9], is designed to discover relations which are not contained in any individual abstract using probabilistic inference. In contrast to this work their approach is based on a collection of hand-built rules, that map linguistic constructs onto a probabilistic reasoning system. Furthermore, it does not make use of any structured knowledge base.

Arguably the most similar work to ours is the work of Lao et al. [10]. As in the current work, the authors use a combination of structured and unstructured knowledge to infer relations between concepts using a sequence of related concepts. They use an open domain, web-scale corpus to train a classifier based on logistic regression with a huge amount of training examples represented by vectors of a very large feature space. However, the requirements of this work, namely a limited amount of training data and a much smaller textual corpus, require a different way of modeling and training.

Table 1 provides an overview of the aforementioned works in comparison to the current approach with respect to different aspects concerning the requirements and used methodologies.

**Table 1:** **Comparison of related work with respect to: use of liguistic information, use of manually designed rules, application in restricted domain, possibility of using sparse training data**

**Work**	**Linguistic**	**Manual**	**Restricted**	**Sparse**
Goertzel et. al (2006) [9]	x	x	x	x
Frijters et al. (2010) [4]			x	(x)
Cohen et al. (2010) [5]				
Lao et al. (2012) [10]	x			
Srinivasan et al. (2004) [6]		x	x	x
Current work	x		x	x

## Methods

### Terminology

In this work an atomic piece of knowledge is considered as a triple (*c*_*i*_,*l*,*c*_*j*_), consisting of a pair of concepts (*c*_*i*_,*c*_*j*_), e.g., (*aspirin*,*inflammation*), and a label *l*, e.g., *may treat*, representing a relation *R*_*l*_ to which the pair (*c*_*i*_,*c*_*j*_) belongs. Furthermore, indirect knowledge connecting two concepts *c*_*s*_ and *c*_*t*_ is defined as a sequence of triples starting with concept *c*_*s*_ and ending in concept *c*_*t*_, where the second concept of each triple must be equal to the first concept of its following triple. Table 2 summarizes the notation used in this article.

**Table 2:** **Summary of the terminology and notation used throughout the manuscript**

**Symbol**	**Explanation**
*c*_*i*_	a concept
*C*	a set of concepts
*l*	a label representing a relation
*R*_*l*_	binary relation with label *l*
(*c*_*i*_,*l*,*c*_*j*_)/triple	a pair of concepts (*c*_*i*_,*c*_*j*_) connected by relation with label *l*
*R*	a set of triples
*G*	the knowledge graph
*P*	a path in *G*
*f*	a feature vector
*E* ^+^/ *E* ^−^	a set of positive/negative examples
***θ***	set of model parameters
***X***	observable variables or observations defined by a model
***H***	hidden or latent variables defined by a model

### Utilized biomedical knowledge sources

Nowadays, plenty of data is freely available and easy to access, but each data source has a different knowledge representation, called a schema. The schema defines how concepts can be described and how they can relate to each other. For structured knowledge sources such as databases the set of relations and concepts as well as their representations are well defined, whereas for unstructured knowledge sources like text this is not the case. Natural language is far more expressive than the schemas of any structured knowledge source because it is not restricted to a fixed set of concepts and relations, but at the same time it is much harder to interpret because natural language can express different pieces of knowledge with the same representation (polysemy), and one piece of knowledge in many different ways (synonymy). In the following, we describe the different biomedical knowledge sources that are used in this work, and how they have been utilized.

#### Unified Medical Language System

The most popular structured knowledge base for biomedical text mining is the *Unified Medical Language System* (UMLS), which consists of 3 different resources, namely the *Metathesaurus*, the *Semantic Network* and the *Specialist Lexicon*. The Metathesaurus is a multilingual vocabulary database which combines knowledge from many different structured knowledge sources. It contains a large amount of biomedical concepts, information about them (e.g., their semantic type, a description, etc.) and how they are related to each other. The Semantic Network comprises a set of semantic types and relations connecting the semantic types to each other. It provides a consistent, semantic categorization of Metathesaurus concepts. The Specialist Lexicon is a general English lexicon consisting of biomedical terms which is not used in this work. The roughly 3 million concepts contained in the Metathesaurus of the 2013AB Release form the basis of the knowledge representation in this work which means that concepts from other knowledge sources have to be mapped to concepts of the Metathesaurus.

#### DrugBank

DrugBank [11] is an open drug and drug-target database. A target of a drug refers to a protein that a drug is able to bind to. DrugBank is not yet part of the UMLS. Therefore, a mapping from DrugBank to UMLS ids is necessary, which can partially be achieved by mapping their respective concept names to each other. By following this approach it is possible to map 1125 targets (proteins or genes) and 2663 drugs from DrugBank to UMLS, which results in a total of 1228 distinct drug-target pairs mapped from all FDA-approved drug-target pairs documented in DrugBank.

#### MEDLINE

As unstructured knowledge source MEDLINE is used. It is the collection of all publication abstracts from all life-science journals indexed by PubMed. MEDLINE is the most widely used, freely available textual corpus for biomedical text mining. Furthermore, an already annotated version of MEDLINE exists. The annotation is performed frequently by the National Library of Medicine (NLM) using the MetaMap program [12], which annotates natural language text with concepts of the UMLS Metathesaurus. The 2012 MetaMapped MEDLINE corpus is used as unstructured textual knowledge source containing all publications until November 18, 2011.

When using the MetaMapped MEDLINE corpus care should be taken. E.g., MetaMap has problems annotating genes with aliases which are common english words such as “impact” or “rare”. In this work we exclude gene/protein annotations for common english words. Furthermore, we only consider annotations of UMLS concepts of the following semantic types or their respective subtypes: Organisms, Clinical Drug, Substances, Sign or Symptom, Anatomical Structure, Molecular Sequence, Body Space or Junction, Body Location or Region, Pathologic Function, Injury or Poisening.

### Dependency trees

**Figure 3:**
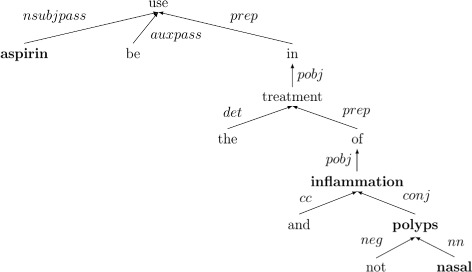
An example of a dependency tree of a sentence. The dependency tree of the following sentence is illustrated: *“Aspirin is used in the treatment of inflammation and not nasal polyps”*.

*Dependency trees* [13] are syntactic constructs of sentences in which each node of the tree represents a token (word or symbol) of the underlying sentence and each arch represents a dependency between two tokens of that sentence. In dependency grammars (DG) the verb always takes the central role of the sentence and is therefore always the root of the tree independent from the rest. Furthermore DGs do not require any ordering of the sentence words and are thus also applicable to languages in which the order of words in a sentence is all the same (e.g., in Czech or Turkish). Unlike phrase structure grammars (constituency grammars) DGs do not explicitly structure sentences into phrases but rely only on dependencies between words in a sentence [14]. An example of a dependency tree is shown in Figure 3.

### Knowledge representation

Integrating knowledge from heterogeneous data sources into one coherent representation (schema) is a complex process. One of the main difficulties is the mapping of the concepts in each data source to each other. This has been done for all used knowledge sources in this work as explained in their respective description. Mapping relations from different knowledge sources to each other is even more complicated and can potentially result in a loss of information. To circumvent this problem relations are kept explicitly in the form they occurred in the sources which results in a huge relation space with a lot of redundancy. The relation space, however, can be reduced by using semantic vector representations for the relations obtained by applying co-occurrence based dimensionality reduction algorithms as will be described later in the description of the encoding.

#### Knowledge graph

In order to find indirect connections between concepts quickly, both structured and unstructured knowledge is represented by a graph. Similar to the work of [10] a directed, edge-labelled graph *G*=(*C*,*R*) is used, comprising a set of concepts *C* as vertices and a set of labelled edges (triples) *R*=*C*×*L*×*C* between them, where *L* denotes the set of all possible relation labels. If there is a pair (*c*_*i*_,*c*_*j*_)∈*R*_*l*_ in one of the knowledge sources, an edge (*c*_*i*_,*l*,*c*_*j*_) is added to *R*. In other words, only if a pair of concepts is known to be in a relation with label *l*, then there is an edge labelled with *l* in *G* connecting this pair of concepts. Note that a triple can occur more than once in *R*, which means that *R* is actually a multiset. A path of concepts *P* in *G* of length *n* is an n-tuple of vertices *P*=(*c*_1_,...,*c*_*n*_), where ∀*i*,1≤*i*<*n*:∃*l*∈*L*:(*c*_*i*_,*l*,*c*_*i*+1_)∈*R*, meaning that there must be at least one edge between the concepts *c*_*i*_ and *c*_*i*+1_ for every *i*.

#### Knowledge extraction

Structured knowledge sources, such as UMLS and DrugBank, already contain labelled relations *R*_*l*_⊆*C*×*C*. The information of all relations *R*_*l*_, i.e. its concept pairs (*c*_*i*_,*c*_*j*_) together with its label *l*, can directly be inserted into the graph by adding all concepts *c*_*i*_ and *c*_*j*_ of all pairs to *C* as vertices and all corresponding triples (*c*_*i*_,*l*,*c*_*j*_) to *R* as edges.

Extracting triples from unstructured, textual data requires a more elaborate strategy. Since MEDLINE, the used textual data, is already annotated with biomedical concepts of the UMLS Metathesaurus, this task reduces to extracting only the relations between concepts found in one sentence. Previous work on relation extraction has shown that the *dependency path* between two concepts in a sentence typically contains all necessary information to recognize a specific underlying relation between them (e.g., [15-18]). A dependency path is a path in a *dependency tree*, which is a syntactic construct of a sentence as explained in the previous section. It is important not to confuse the notion of dependency path, which are edges in *G*, with the notion of a path in the knowledge graph *G*.

Triples are only extracted from sentences when a pair of concepts, or more precisely their headwords in the dependency tree, connected by a dependency path are found that contains at least one verb form. If the dependency path does not contain any verb form, it is assumed that there is no relation present in this sentence. On the other hand, if two or more verb forms are found on the dependency path which are part of two distinct sub-sentences connected by some conjunction, it is assumed that there is no direct relation in the sentence between such concept pairs present and these triples are discarded as well. Furthermore, conjunction and apposition edges are removed from the dependency paths together with their head words because in most cases they represent simple enumerations which do not effect the semantics of the relation being expressed between the two concepts in question. If there is a negated noun or verb form present on the dependency path, the whole path will be treated as negated as well. Furthermore, there is also the issue of extracting triples connected by very long dependency paths. Long dependency paths can be very unspecific and confusing, and they are more likely to contain parsing errors. Moreover, such paths occur usually very rarely in the corpus which makes them hard to interpret when using statistical methods. After manual inspection a maximum length of 6 was chosen to prevent that. Once a pair of concepts (*c*_*i*_,*c*_*j*_) is extracted from a sentence together with its post-processed dependency path *p*, a triple (*c*_*i*_,*p*,*c*_*j*_) can be inserted into the knowledge graph *G* the same way as for structured knowledge. Note that there is no mapping from the extracted dependency paths to any specific predefined relation label. Therefore, every possible dependency path can be viewed as a single relation.

As an example of this procedure it is possible to extract the following triples from the sentence shown in Figure 3:

- $\big (\textbf {aspirin}, \overset {nsubjpass}{\rightarrow } use \overset {prep}{\leftarrow } in \overset {pobj}{\leftarrow } treatment \overset {prep}{\leftarrow } of \overset {pobj}{\leftarrow }, \textbf {inflammation}\big)$
- $\big (\textbf {aspirin}, neg\rule {1ex}{.4pt}\overset {nsubjpass}{\rightarrow } use \overset {prep}{\leftarrow } in \overset {pobj}{\leftarrow } treatment \overset {prep}{\leftarrow } of \overset {pobj}{\leftarrow }, \textbf {nasal polyps}\big)$

In the second triple the *conj*-sequence is removed from the path and the overall path is negated because it contains a negated noun phrase.

For both unstructured and structured triples (*c*_*i*_,*l*,*c*_*j*_) in the knowledge graph, there is always an inverse triple (*c*_*j*_,*l*^−1^,*c*_*i*_). During extraction these inverses are excluded and only one triple (*c*_*i*_,*l*,*c*_*j*_) is added to the graph to avoid including redundant information. However, during path search we also consider triple (*c*_*j*_,*l*^−1^,*c*_*i*_) to be existent.

**Figure 4:**
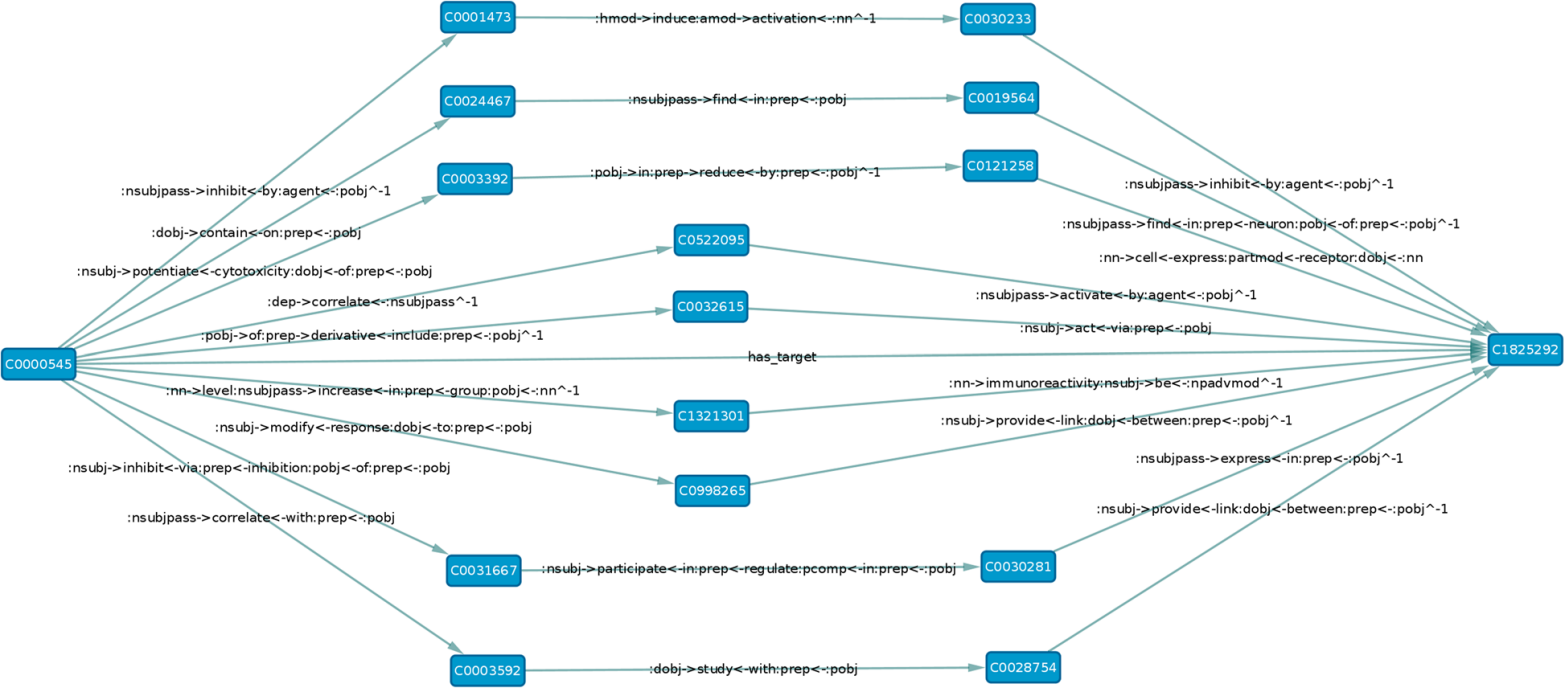
A sub-graph of the knowledge graph. This sub-graph consists of example paths connecting the two concepts *C*0000545 (Eicosapentaenoic Acid) and *C*1825292 (FFAR1 gene) which are part of the *has target* relation.

An example sub-graph of the resulting knowledge graph can be found Figure 4.

### Modelling

In order to discover that a pair of concepts *c*_*s*_ (source) and *c*_*t*_ (target) is in a relation *q* in question using indirect connections between them, i.e., paths in *G* of length greater than 2, a model must be trained to recognize typical graph path patterns for *q* from positive and negative training pairs. Direct connections are excluded to avoid explicit inference of the relation in question. During the application of the model, a set of graph paths extracted between *c*_*s*_ and *c*_*t*_ are presented to the model which in turn calculates a confidence score between 0 and 1 of assigning label *q* to the concept pair (*c*_*s*_,*c*_*t*_) in question. In the following we describe these steps in more detail.

Two types of models are considered for modelling the problem of discovering a relation between a pair of concepts given a set of paths connecting them. The first type of modelling directly extracts features from the set of paths between a pair and uses the resulting feature vector as input for any kind of vector classifier. This method will be referred to as the pair-based approach. A second approach is based on a model that assigns confidence scores to graph paths rather than to the pairs themselves. Given all graph paths and their respective scores between two concepts, the final confidence score for the concept pair is calculated by averaging its best *k* graph path scores. The problem of considering all paths and averaging them to form the final score is that nearly all graph paths are uninformative with respect to the relation that exists between the source and the target concept. This would introduce a lot of noise and disturb the resulting scores.

#### Relation label encoding

Encoding a graph path *P*=(*c*_1_,...,*c*_*n*_) requires an encoding of each connection (*c*_*i*_,*c*_*i*+1_) in *P* as a feature vector $f_{(c_{i},c_{i+1})}$, resulting in a sequence of feature vectors of length (*n*−1) which is used by the model for training of inference, respectively. $f_{(c_{i},c_{i+1})}$ is defined as the sum of all feature vectors of each relation label *l* occurring between *c*_*i*_ and *c*_*i*+1_ (see Equation 1). Feature vectors for all possible relation labels *l* can be created in different ways. The following two sections explain how this is achieved in this work.

(1) $$ f_{(c_{i},c_{i+1})} = \sum_{(c_{i},l,c_{i+1}) \in R} f_{l}  $$

##### One-of-N encoding.

The simplest way of encoding a relation label *l* is the one-of-N encoding, where only the *l*-dimension of the feature vector has value 1 and all others are 0, as the name of suggests. This encoding, however, is very poor because it does not take any semantic similarities or even synonymy among the relation labels into account, which leads to an explosion of the feature space growing as large as there are different relations. Especially for unstructured relation labels (i.e., dependency paths) there are many ways of expressing the same underlying relation, resulting in a lot of redundancy. With a large number of training examples this can be handled by the model, but if training examples are sparse, there is a need of encoding relations in a much smaller semantic feature space or otherwise the model will overfit to the training data.

##### Semantic encoding.

Mapping relation labels into a semantic space has already been done in other studies such as the work of Yao et al. [19]. Extracting semantic vectors for words co-occurring in documents is a well studied problem and thus, there are numerous algorithms that solve this task. Examples are *latent semantic analysis* (LSA, [20]), *reflective random indexing* (RRI, [5]), *the generalization of principle component analysis* (gPCA, [21]) or *latent Dirichlet allocation* (LDA, [22]). The basic idea for constructing a semantic space of relations is to consider a pair of connected vertices, i.e. concepts, (*c*_*i*_,*c*_*j*_) in *G* as a document *d*_*i*,*j*_ and the label of each edge between them as a word occurring in *d*_*i*,*j*_. Using this transformation for all connected concept pairs of *G*, the above mentioned algorithms can be used natively to construct semantic feature vectors of a specified size for each relation label or in case of LDA even for each concept pair at the same time. In the experiments LDA is used because its underlying model fits well to this problem.

By transforming pairs of concepts to documents and relation labels to words, LDA’s latent topics can be considered as the “true” but hidden relations between a pair of concepts. Each true relation has many different forms of representations in natural language text or databases. At the same time, the number of possible true relations between a pair of concepts is usually very low and in many cases even one, thus they are also very sparse. These two aspects can be reflected in LDA by setting the hyper-parameters of the model to something well below 1. The idea of modeling relations with LDA was already investigated in a similar form by Yao et al. [23].

In case of using LDA features, we define *f*_*l*_ as the conditional probability distribution over all possible latent topics *t* given relation label *l*:

(2) $$\begin{array}{*{20}l} {f_{l}^{t}} &= p(t | l)  \\ p(t|l) &\propto p(t) \cdot p(l|t), \end{array} $$

where ${f_{l}^{t}}$ is the value of the *t*-th dimension of *f*_*l*_. *p*(*t*) and *p*(*l*|*t*) are directly extracted from the trained LDA model. Furthermore, in case of LDA, all pair feature vectors $f_{(c_{i},c_{j})}$ are also normalized after summing over all feature vectors of labels occurring on edges between (*c*_*i*_,*c*_*j*_).

In order to validate that LDA is able to learn semantic vector representations of relations, the 15 most occurring dependency paths of the 100 semantically most similar dependency paths to the *may treat* and *has target* relation were extracted. The resulting sets of relations are shown in Table 3. From the examples, it can be seen that most of the extracted dependency paths for both of the relations are actually textual representations of them, which supports the claim that semantic vectors of relations can indeed be learned using LDA.

**Table 3:** **The 15 most popular relations taken from the 100 semantically closest relations to the** ***has target*** **and** ***may treat*** **relation**

**Relation**	**Most similar relations**
*has target*	
*may treat*	

#### Pair-based approach

For pair-based classification a vector classifier must be trained which takes as input a feature vector. Similar to the previous work [10] a logistic regression model is used in this approach. Given a set of graph paths $\mathfrak {P}_{c_{s},c_{t}}$, the feature vector $f_{c_{s},c_{t}}$ for (*c*_*s*_,*c*_*t*_) is defined as the normalized sum of the feature vectors representing the paths $P \in \mathfrak {P}_{c_{s},c_{t}}$. The feature vector of a path *P*=(*c*_1_,...,*c*_*n*_) is calculated from its corresponding sequence $f_{(c_{i},c_{i+1})} \in \mathbb {R}^{N}$ of feature vectors by transforming their outer product which is an (*n*−1)-dimensional tensor into a vector representation.

(3) $$\begin{array}{*{20}l} f_{c_{s},c_{t}} &= \frac{\sum_{P \in \mathfrak{P}_{c_{s},c_{t}}} f_{P}} {\left\vert \sum_{P \in \mathfrak{P}_{c_{s},c_{t}}} f_{P} \right \vert} \end{array} $$

(4) $$\begin{array}{*{20}l} f_{P} &= \pi\left(f_{(c_{1},c_{2})} \otimes \cdots \otimes f_{(c_{n-1},c_{n})}\right) \end{array} $$

The resulting vector consists of *N*^*n*−1^ dimensions, where each dimension corresponds to a tuple in {1,2,⋯,*N*}^(*n*−1)^ which represents a position in the former tensor. The following equation is an example of transforming the outer product of two 4-dimensional vectors ***u*** and ***v*** into a vector representation.

$$\begin{array}{@{}rcl@{}} \pi\left(\boldsymbol{u}\otimes\boldsymbol{v}\right) &=& \pi\left(\boldsymbol{u} \boldsymbol{v}^{T} \right) = \pi \left(\left[ \begin{array}{lrrrr} u_{1} v_{1} & u_{1} v_{2} & u_{1} v_{3} & u_{1} v_{4} \\ u_{2} v_{1} & u_{2} v_{2} & u_{2} v_{3} & u_{2} v_{4} \\ u_{3} v_{1} & u_{3} v_{2} & u_{3} v_{3} & u_{3} v_{4} \\ u_{4} v_{1} & u_{4} v_{2} & u_{4} v_{3} & u_{4} v_{4} \\ \end{array} \right] \right)\\ & =& \left(\begin{array}{lrrrr} u_{1} v_{1} \\ u_{1} v_{2} \\ u_{1} v_{3} \\ u_{1} v_{4} \\ u_{2} v_{1} \\ \cdots \\ u_{4} v_{3} \\ u_{4} v_{4} \end{array} \right) \end{array} $$

One problem of using this type of encoding is the exponentially growing feature space depending on the maximum path length *m*. The pair-based approach in conjunction with the plain one-of-N encoding has a feature space in which each dimension corresponds to a sequence of relation labels. The model learns which of those relation label sequences are characteristic for the relation the model is being trained on which is reflected by a high positive weight for the corresponding dimension. This is very useful for the interpretation of what the model has learned because the extraction of highly weighted relation label sequences from the trained model is very easy.

#### Path-based approach

For path-based classification a binary sequence classifier must be trained, which takes as input a sequence of feature vectors $f_{(c_{i},c_{i+1})} \in \mathbb {R}^{N}$ (see Equation 1) constructed from the graph path *P*=(*c*_1_,...,*c*_*n*_) in question and outputs a confidence score. This can be modelled by using any kind of vector-classifier which takes as input a feature vector of length (*m*·*N*), if the maximum possible length *m* of a sequence is known, or it is possible to use a proper sequence classifier. For the former *logistic regression* and for the latter a combination of two *hidden markov models*, one trained on positive example paths for *q* (pHMM) and the other only trained on negative example paths (nHMM), were chosen. In case of the HMMs a path *P* is applied to both HMMs during inference and the probability of sequence *P* given the respective HMM is computed. The confidence score of assigning label *q* to path *P* is finally calculated by combining the two probabilities in the following way:

(5) $$\begin{array}{*{20}l} p_{HMM}(q|P) &= \frac{p(P,q)}{p(P,q)+p(P,\neg q)}  \\ &= \frac{p(P|q)}{p(P|q)+p(P| \neg q)} \end{array} $$

where *p*(*P*|*q*)=*p*_*pHMM*_(*P*) denotes the probability of *P* calculated by the positive HMM and *p*(*P*|¬*q*)=*p*_*nHMM*_(*P*) the probability calculated by the negative HMM, assuming *p*(*q*)=*p*(¬*q*), which is a strong assumption. In reality *p*(¬*q*)>>*p*(*q*), however, this would put too much weight on the outcome of the negative HMM. In practice, we are typically more interested in finding a good ordering of tuple candidates for a relation instead of real probabilities for single candidate tuples based on the output of both models.

The advantage of this path-based approach over the pair-based model is that the feature space grows only linearly with the maximum path length *m* in the case of logistic regression and it is even constant with increasing *m* for the HMM approach.

### Graph path discovery

To extract paths for a concept pair (*c*_*s*_,*c*_*t*_) to a maximum path length *m*, a bidirectional search [24] is performed, i.e., searching is done by starting in both vertices *c*_*s*_ and *c*_*t*_ until a maximum path length of $\left \lfloor \frac {m+1}{2} \right \rfloor $ from each side is reached. Although search is still exponential in time and space complexity, it requires only the square root of resources compared to naive search from the source to the target vertex. Finally, similar to the work of [10], the probability of expanding the search to a neighbor vertex *c*_*j*_ from the current vertex *c*_*i*_ is given by the following formula:

(6) $$ p_{\text{explore}}(c_{j}|c_{i})= min\left(1,\frac{\sqrt{h+|N(c_{i})|}}{|N(c_{i})|}\right)  $$

where *N*(*c*) denotes the set of neighbors of vertex *c* and *h* is a big number (e.g., 100,000 in this work). Usually the number of neighbors is not very high, which means that in most cases every neighbor will be explored.

Example paths of different sizes (2-4) can be seen in Figure 4, which illustrates a sub-graph of the knowledge graph containing paths between a drug and its target.

### Training

Models are trained using distant supervision, which assumes that paths between a pair of concepts of relation *q* are representing *q* and can therefore be considered positive training examples for *q*. Even though this assumption is strong it has been shown to be very effective in previous studies [25,26].

Training examples for a specific relation *q* can directly be extracted from its relation *R*_*q*_⊂*C*×*C* contained in at least one of the structured knowledge sources (e.g., DrugBank and/or UMLS). A model for relation label *q* is trained with a set of positive training examples $E_{q}^{+} \subseteq R_{q}$ and a set negative training examples $E_{q}^{-} \subset C \times C$, which is constructed from $E_{q}^{+}$ by pairing all source concepts of $E_{q}^{+}$ with a random target concept of $E_{q}^{+}$, ensuring that $R_{q} \cap E_{q}^{-} = \text {\O }$. By using the same concepts in both the positive and the negative training set, it is ensured that the model does learn only about the paths between the pairs rather than also learning characteristics about the different concepts of the two training sets.

Given a set of positive ($E_{q}^{+}$) and negative training examples ($E_{q}^{-}$) for relation label *q*, a graph path classifier is trained on all extracted graph paths for each concept pair of $E_{q}^{+}$ and $E_{q}^{-}$. For HMMs, standard EM training (*Baum-Welch algorithm*) is applied, and for logistic regression, training is performed using gradient ascent on the likelihood function using LBFGS with L2-regularization.

As described previously, only very few of the graph paths extracted between concepts of a positive pair are real indicators for the relation label *q*. This is a problem when training the path-based classifier model, because it means that most of the extracted positive example graph paths, which are the paths between concepts of a positive concept pair are actually negative or noisy examples. To deal with this problem most of the noise from the positive path training examples can be removed as follows. First, a model is trained on the initial, noisy examples. Subsequently, the trained model is used to score all positive graph paths in order to eliminate noisy paths by only keeping those positive graph paths that were scored higher than a specific threshold (e.g., 0.5 in our case). In turn, a completely new classifier model can be trained on the pruned set of positive graph paths and the original set of negative graph paths. This procedure can be repeated several times, though once was already enough in our experiments. Training the path-based HMM classifier in such a way has shown to be more effective in the conducted experiments and a clearer separation between the distribution of the confidence scores of the positive compared to the negative training examples was observed.

Finally, for learning semantic relation vectors the LDA model was trained using the efficient sparse stochastic inference algorithm developed by [27], which is particularly useful when dealing with huge amounts of training data.

## Results and discussion

### Graph generation

**Figure 5:**
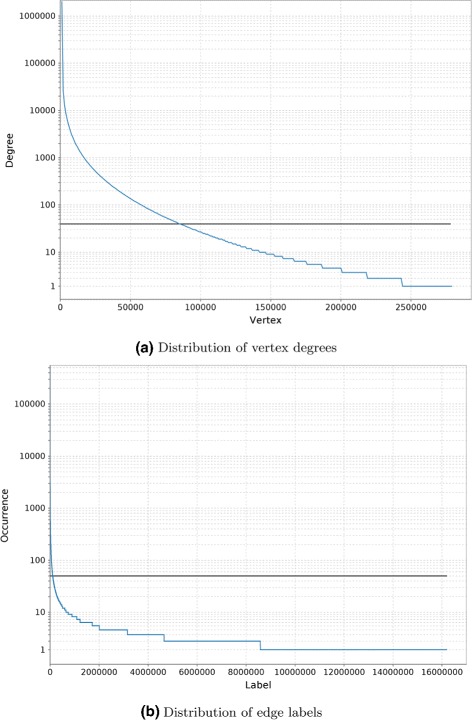
Distribution of vertex degree and edge labels in unpruned, unstructured part of the knowledge graph, in log-scale. Figure **(a)** shows the distribution of vertex degrees. Similarly, Figure **(b)** shows the distribution of edge labels.

For the already annotated MEDLINE corpus of 2012, triples were extracted by extracting dependency paths between two annotated concepts in each sentence. ClearNLP [28] was used for dependency parsing, because it is very fast and provides existing models trained on medical text. The resulting set of triples was stored in a titan [29] graph database. During extraction only dependency paths of length up to 6 were considered. The resulting graph contains 278,061 vertices (i.e., concepts) with an average degree of 600 in- and outgoing edges, resulting in 83 million edges (i.e., extracted triples) of around 16 million different labels (i.e., dependency paths), where each label thus occurs on average 5.2 times. In total, 29.7 million pairs of vertices are connected to each other. Both vertex degrees and edge label occurrences follow a very heavy tailed distribution (see Figure 5), i.e., most of the vertices and edge labels only occur very scarcely. Because there is so little data for those concepts and dependency paths, there is no value in keeping those for statistical learning methods. Therefore, the graph was pruned at a total concept occurrence of at least 40 for vertices and a total label occurrence of at least 50 for edges, after manual inspection of the occurrence statistics (see Figure 5). The pruned, unstructured part of the knowledge graph contains 84,635 vertices and around 39 million edges with 104,953 different labels between around 9 million connected concept pairs. Another 2.8 million pairs for relations stemming from UMLS and DrugBank were added to the graph as edges, but no new concepts were introduced, because the graph would have grown too large if all concepts of the UMLS would have been included as vertices.

### Path search

Finding paths between two concepts in such a highly connected graph is computationally challenging, because search time increases exponentially with the specified maximum path length. Thus, given a pair of concepts (*c*_*s*_,*c*_*t*_), only paths up to a certain maximum length *m*=4 were extracted by performing a bidirectional search. During search, synonyms^c^ of concepts on the currently explored path were not allowed to be explored in the next step. One problem that arises is the fact that some vertices, called hubs, are connected to many concepts (e.g., the concept of *cell*), which lets the search space explode if hubs are explored. However, paths running through such hubs can be considered less informative than paths running through scarcely connected vertices, because hubs are very general concepts. Therefore, to avoid this problem and to make the search algorithm faster, highly connected vertices (degree greater than 100,000) are excluded from search. Furthermore, in some cases the number of all possible paths gets very large even with a maximum path length *m*=4. Therefore, search time was limited to 40 seconds per pair.

### Datasets and training

**Figure 6:**
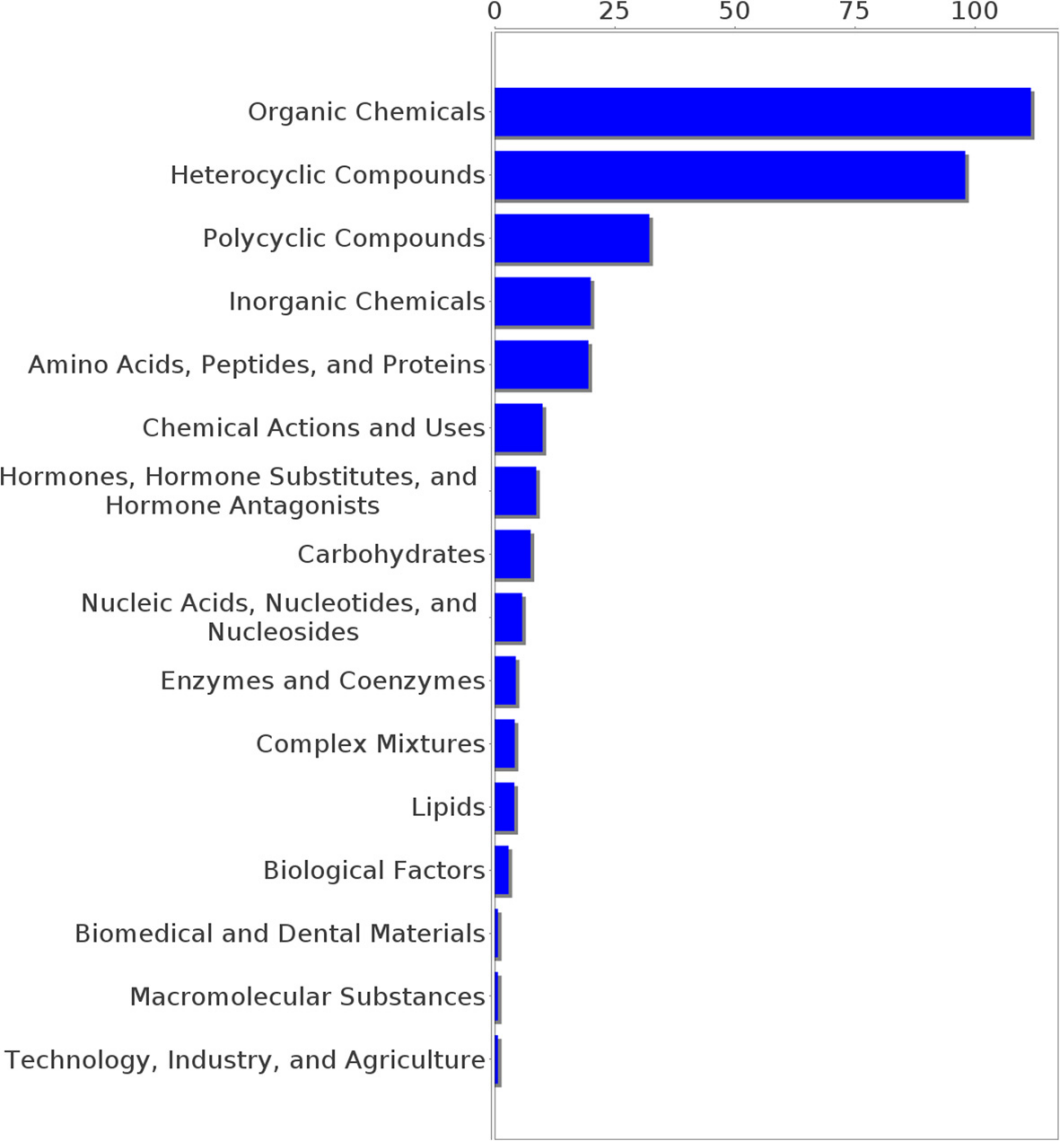
Distribution of drug types in the *may treat* dataset. The distribution of the drug types occurrences in the *may treat* dataset is shown.

**Figure 7:**
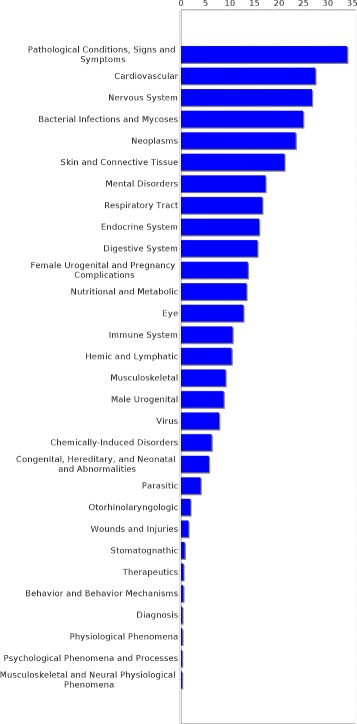
Distribution of disease types in the *may treat* dataset. The distribution of the disease types occurrences in the *may treat* dataset is shown.

**Figure 8:**
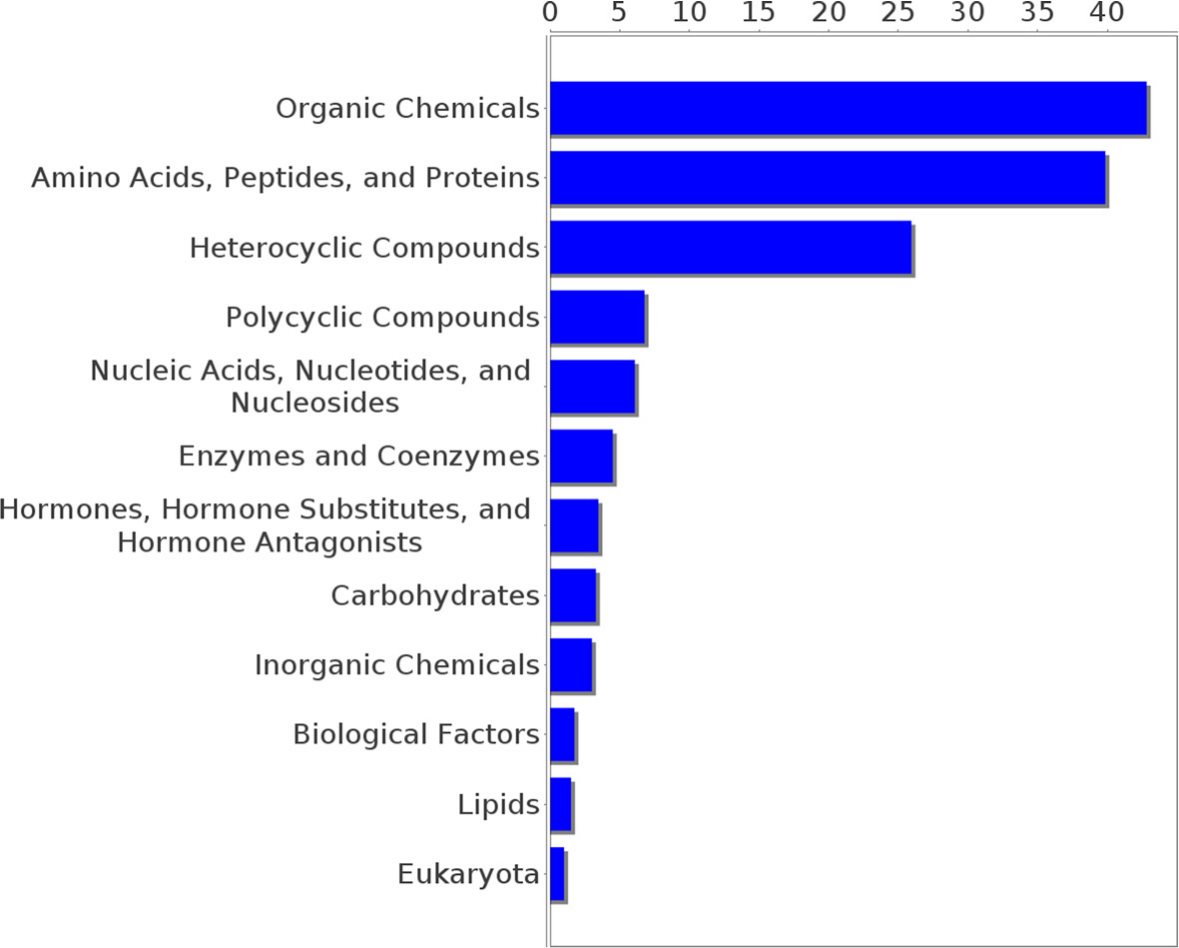
Distribution of drug types in the *has target* dataset. The distribution of the drug types occurrences in the *has target* dataset is shown.

**Figure 9:**
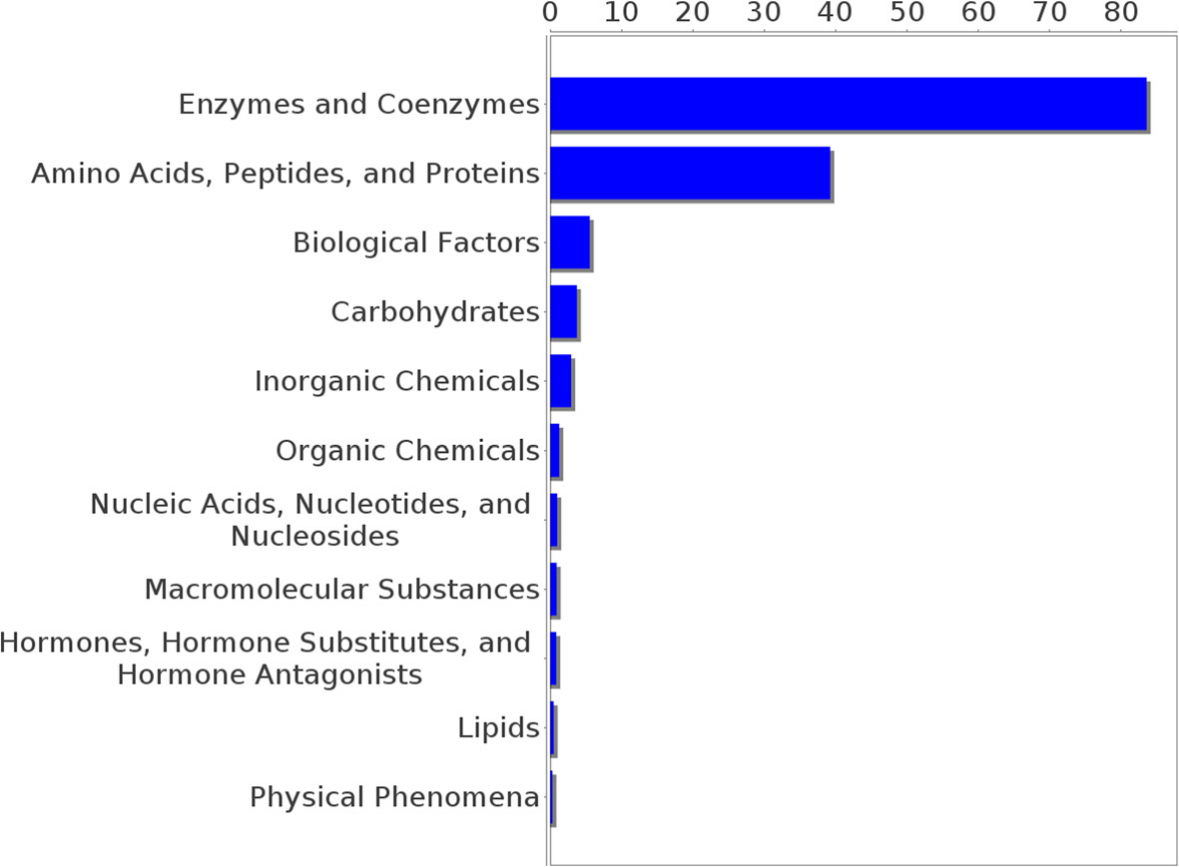
Distribution of target types in the *has target* dataset. The distribution of the target types occurrences in the *has target* dataset is shown.

Experiments were conducted on two different datasets, pertaining to two different relations, though the approach is applicable for learning any new relation, provided that it comprises concepts from the UMLS metathesaurus. The first dataset contains 438 concept pairs of the *may treat* relation taken from the UMLS. It was constructed with two restrictions in mind. First, it was ensured that no drug or disease concept occurred more than once in the whole dataset and second, every concept in that dataset had to be part of the pruned graph. The former restriction assured that the diseases are not dominated by one disease type (e.g., neoplasms, cardiovascular diseases etc.), but that many types of diseases are represented proportionally in each category. The latter restriction was made because for the extraction of paths the pair of concepts in question has to be part of the graph. Figures 6 and 7 show the distribution of drug and disease types, respectively, contained in that dataset. The second dataset consists of 744 pairs of the *has target* relation extracted from DrugBank and mapped to UMLS. As for the *may treat* dataset it was ensured that all concepts are part of the pruned knowledge graph but multiple occurrences of one concept were allowed. Figures 8 and 9 show the distribution of drug and disease types, respectively, contained in that dataset. Both datasets were constructed by extracting all concept pairs that are contained in the respective relation from the UMLS and afterwards the pairs were filtered with the aforementioned restrictions in mind. Negative examples were constructed as described in the previous section. Note that ensuring the exclusiveness of positive and negative examples can lead to a slightly smaller set of negative examples. The used datasets are publicly available and can be found as Additional file 1.

During path extraction, edges labelled with *may treat* or *has target*, respectively, and the sibling (SIB) label were ignored. The sibling relation expresses that two concepts have the same parent concept. We found that sibling concepts usually have very similar relations. For example, drugs of the same family often treat the same diseases. Thus paths like $c_{s}\xrightarrow {SIB} c_{x} \xrightarrow {\text {\textit {may treat}{}}}c_{t}$ and $c_{s}\xrightarrow {SIB} c_{x} \xrightarrow {\text {\textit {has target}{}}}c_{t}$ occurred frequently as positive training examples for the *may treat* and *has target* relation, respectively. Those obvious connections could potentially distort the results. If not stated otherwise, concept pairs, for which no paths of the specified lengths could be found, were excluded in the experimental evaluation. The number of exclusions depends on the maximum allowed path length. E.g., only around 36*%* of all *has target* pairs have paths of length 2 (i.e., direct connections). Finally, all models and training algorithms mentioned in the previous section were implemented using the FACTORIE toolkit [30], version 1.0.0-RC1.

### Results

All results were obtained by evaluating the proposed models on the datasets using 10-fold cross validation, if not stated otherwise. Classification performance was evaluated by the area under the curve (AUC) value of the ROC-curve, a common classification evaluation method for information retrieval systems. Other evaluation metrics based on the precision of the system are not useful in this context because the datasets consist of an equal number of positive and negative examples, which is not the case in reality, where there are much more negative example pairs (e.g., consider all possible drug-disease combinations from which only small fraction is in a *may treat* relation). Sensitivity (*true positive rate*) and specificity (*false positive rate*), which make up the ROC curve, are independent of the prior distribution of positive and negative examples. Special focus should be given to the steepness of the ROC curves at their beginning, because it can indicate that the models learned some very characteristic path-patterns for a relation (e.g., see Tables 4 and 5). Note that example pairs for which no paths were found were excluded in the evaluation of the experiment.

**Table 4:** **Example plain path patterns of length 3 for the** ***has target*** **relation with high feature weights learned by pair-based logistic regression**

**Highly weighted feature**	**Explanation**
	The substance is induced into something, in which the target (gene/protein) is expressed.
	Some levels of the substance were measured in something that is associated with the target.
	Some levels of the substance were measured in something, to which the target is susceptible.

**Table 5:** **Example plain path patterns of length 3 for the** ***may treat*** **relation with high feature weights learned by pair-based logistic regression**

**Highly weighted feature**	**Explanation**
	The drug treats something (e.g., a symptom) that is diagnosed together with the disease.
	The drug suppresses something that is increased by the disease.
	The drug’s behavior mimics the effect of something which seems to have an effect on the disease.

#### Comparison of models and feature types

Table 6 shows the performance of the different models on the two datasets encoded with both plain one-of-N and LDA features using only paths of length 3. The first finding is that the pair-based approach consistently outperforms the path-based approach, for which logistic regression seems to be the better model. This outcome can be explained by considering the fact that the pair-based approach relies on a much larger feature space (exponential in the maximum path length *m*) compared to the two path-based approaches (linear and constant in *m*), providing more information to the model that seems to be necessary for sophisticated classification.

**Table 6:** **Results using different models and encoding (path length 3)**

**Dataset**	**Model**	**AUC**	
		***Plain***	***LDA***
*may treat*	LR_pair_	0.61	**0.73**
	LR_path_	0.62	0.71
	HMM_path_	0.48	0.68
*has target*	LR_pair_	**0.78**	0.72
	LR_path_	0.64	0.67
	HMM_path_	0.59	0.60

*LR* logistic regression, *HMM* Hidden Markov Model, path - path- based feature encoding; pair - pair-based feature encoding.

With bold, the best AUC values for Plain and LDA are highlighted.

Results on the *has target* dataset show that the AUC can reach up to 0.8 compared against a random baseline with 0.5 AUC, which picks a class label at random. Thus, our approach demonstrates its ability to recognize the “signal” of the relation.

**Figure 10:**
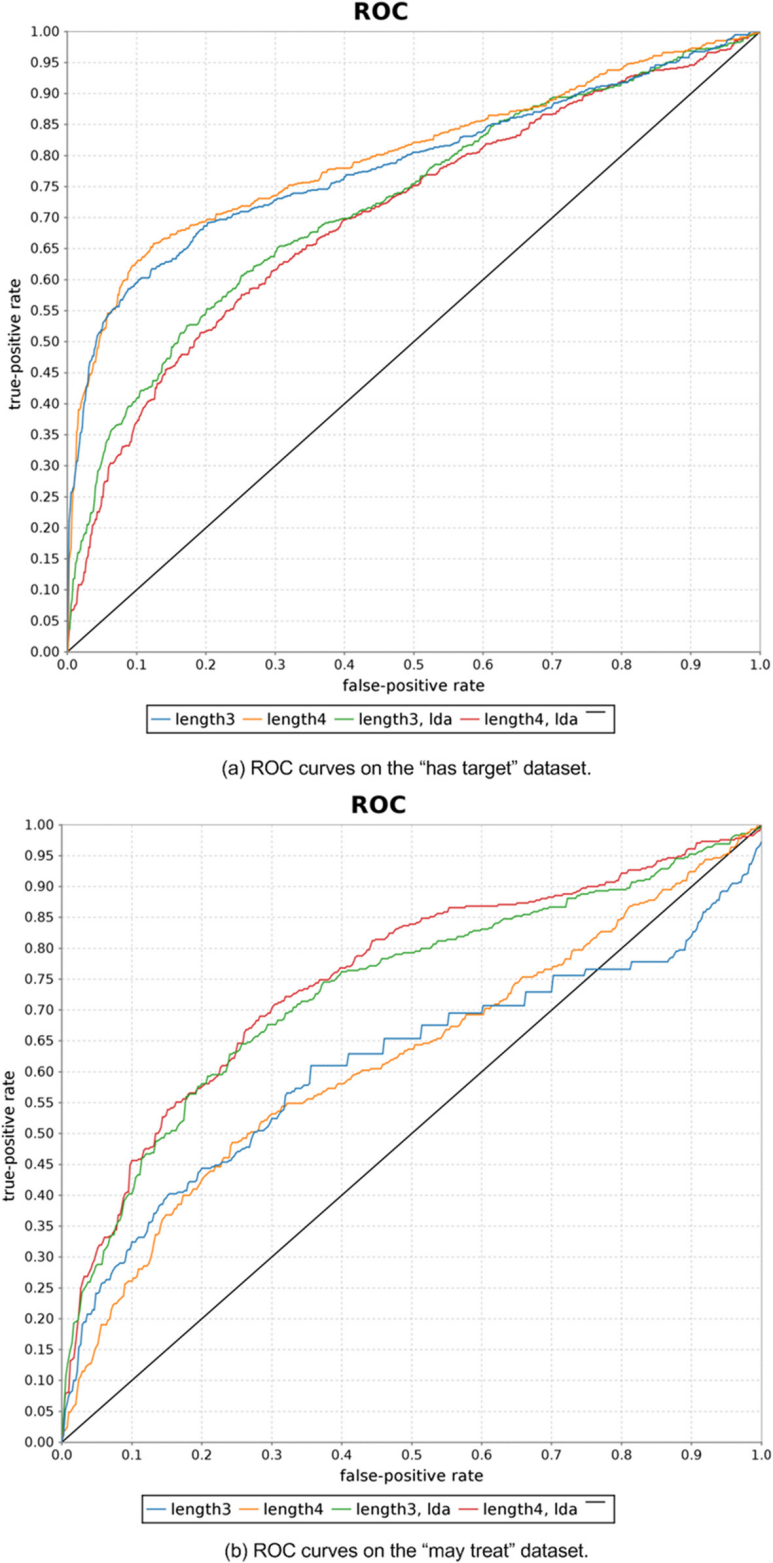
ROC curves for the *has target* and *may treat* datasets. Figure **(a)** shows the ROC curves produced based on the validation conducted on the *has target* dataset. Similarly, Figure **(b)** shows the ROC curves produced based on the validation conducted on the *may treat* dataset.

Another interesting finding is that the ROC-curves of the pair-based approach are very steep at the beginning up to a recall level of around 0.6 (see Figure 10). In particular, this can be observed, when using plain features on the *has target* dataset. This indicates that there are some common path patterns which can be learned by the model and be used to infer the *has target* relation. Table 4 shows some highly weighted example patterns learned by the model.

**Figure 11:**
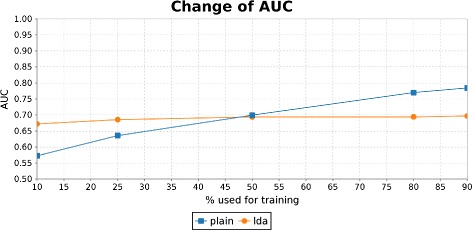
Change of classification performance using different amounts of training data. The difference in classification performance is plotted when a varying number of training examples is used for the LDA and the plain feature extraction method respectively.

The impact of the different feature types cannot directly be inferred from Table 6. In the *may treat* dataset the LDA encoding seems to help a lot, but on the *has target* dataset, which contains about double the amount of training examples, it does not. To evaluate the impact of the different feature types, experiments with different amounts of training examples of the *has target*-dataset were conducted. The results were obtained using cross-validation and are presented in Figure 11.

Models trained with one-of-N features depend highly on the amount of supplied training data, whereas models trained on examples with LDA features do not. This shows the potential of encoding relations with LDA, as it transfers them into a much lower-dimensional, semantic space, which reduces the amount of required training data. However, it can also be seen that information is lost in that process which explains the lower performance achieved with a larger training set.

#### Impact of path length

In order to evaluate the impact of the maximum path length on the overall performance on the two datasets, experiments were conducted with the pair-based logistic regression model on all paths up to length 3 and 4, respectively. Table 7 shows that using paths of length 4 does not improve the overall performance on the classification task. This could be due to the fact, that with increasing maximum length the number of additional informative paths gets lower, while the total number of extracted paths gets exponentially bigger and so does the noise and feature space. This can lead to overfitting of the model to the training data, because training data is too sparse compared to the large feature space. Figure 10 summarizes the results of the previous two sections by showing the ROC-curves for the two datasets with different feature types and maximal lengths.

**Table 7:** **Impact of maximum path lengths using pair-based logistic regression**

**Dataset**	**Length**	**AUC**	
		***Plain***	***LDA***
*may treat*	3-3	0.61	0.73
	3-4	0.62	0.75
*has target*	3-3	0.78	0.72
	3-4	0.80	0.70

The notation *n-m* means that only paths of minimum length *n* and maximum length *m* are allowed.

#### Temporal impact of established knowledge

From the previously presented results it is not clear how much the classifiers depend on the maturity of the respective knowledge that relates two concepts. It might be the case that the trained models are only able to discover relations between pairs that are known to be in that relation for a long time, which should be reflected in the amount of literature that implicitly relates these concepts to each other. A validation dataset consisting of 42 drug repositioning cases, that were collected manually from literature, has been used to validate the performance of the classifier trained on the entire *may treat* dataset in that respect. Drug repositioning refers to the application of known drugs to new diseases. It is an interesting use case scenario because these drugs and diseases are usually well known and described in the literature individually, even though their connection might have only been established recently.

**Figure 12:**
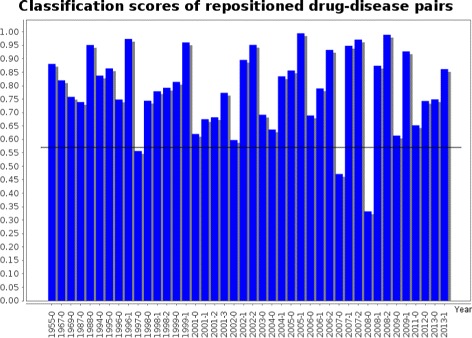
Confidence scores of trained *may treat* classifier using LDA features on a drug repositioning dataset. The figure shows the results of the application of the trained *may treat* classifier, to a drug repositioning dataset, with real case studies of repositioning collected from the period 1955 to 2013. The average classification score of negative training pairs is included as baseline at 0.57.

The resulting scores are ordered by year of FDA approval and are presented in Figure 12. The first finding is that the scores seem to be independent from the year of approval. The classifier is able to classify even most of the very recent repositioning cases with a high score. These results show that recently established knowledge can be discovered by this approach and suggest that even the discovery of new knowledge might be possible. It is noticeable that the confidence scores of the classifier are in general very high on the repositioning dataset, considering that the average classification score of negative pairs for this classifier is 0.57 with only little variation among the scores of those negative examples.

Note, that 4 of the 42 examples in the drug repositioning dataset are also contained in the training set for the *may treat* relation, namely 1967-0, 1999-0, 2001-3, 2002-0. However, they account for less than 10% and therefore do not affect the qualitative observations of this experiment.

#### Using indirect connections for relation discovery

**Figure 13:**
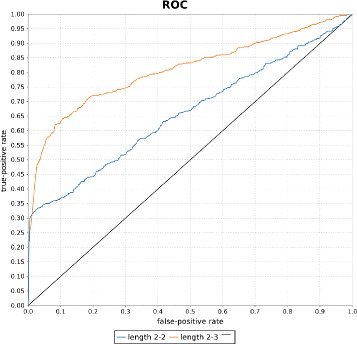
ROC curves using a varying number of path lengths. The figure shows the ROC curves for using paths of only length 2 and paths of both length 2 and 3 on the *has target* dataset.

In many approaches to knowledge discovery (e.g., for database curation), only direct mentions of two concepts in one sentence are being considered to assert a specific relation between two concepts. This approach can be reflected in our setting by only considering paths of length 2 (i.e. only direct connections), which were excluded for all previous experiments. The exclusion from the previous experiments follows the rationale that this approach aims to find new, unknown facts, based on indirect connections between concepts. Furthermore, the problem of only using direct connections is that only around 36*%* of the *has target* pairs and 46*%* of the *may treat* pairs have direct connections in the graph, which means that it is not possible to classify more than those correctly. The improvements of adding indirect connections as features can be seen in Figure 13. By using indirect connections almost twice the number of positive examples can be ranked highly compared to the case of only using direct connections. Note that pairs of the *has target* dataset which do not have any connections of length 2 or 3, respectively, were also included in this experiment to illustrate the recall improvements when indirect connections are included as features.

### Discussion

The results of the experiments show the potential of the suggested approach. By considering indirect knowledge, models can be trained to discover hidden relations between concepts that cannot be extracted directly. This has several potential applications. One application is the curation of databases, where new knowledge can be inferred by combining already established facts. Another example is the inference of completely new knowledge, like the task of drug repositioning. A model can learn from examples typical patterns of indirect connections between a drug that has been repositioned to a disease. This requires a simple adoption of the current approach to only consider knowledge that has been established prior to the first mention of a drug being a potential repositioning candidate for a disease. Moreover, a trained model can be used to find interesting indirect connections between two concepts with respect to a specific relation provided that a curated gold standard of this information can be generated. Predefined relations are not necessarily a prerequisite of the approach, but only a set of concept pairs is needed to learn characteristic path patterns. In general, it is very simple to integrate new knowledge sources or learn path patterns of any relation in the knowledge graph. The simplicity in the design of the approach is a great advantage that offers a lot of flexibility regarding the knowledge sources that can be included in the knowledge graph, and which has many potential applications.

However, besides all the positive aspects of the suggested approach, there are also problems some of which are not easily solvable whereas others could be resolved in future work. The construction of the unstructured knowledge graph consists of several stages in which errors occur that accumulate in the resulting graph. For example, the concept annotation using MetaMap is in some cases very poor, especially for genes. The simple word “an” gets very frequently annotated with the DIAPH3 gene which has the alias AN. Other examples include the word “impact” annotated with the IMPACT gene, and “rare” with the Retinoic Acid Response Element (short alias RARE). Moreover, in scientific articles sentences are more complicated than in other texts because they tend to consist of many nested sub-sentences which makes the linguistic analysis, especially for the dependency parser, more difficult.

Another issue is incomplete knowledge. For example, the extraction of information from text does not take co-references into account. The same problem exists for the used structured data sources. It could not be verified that the use of relations from UMLS and DrugBank has a positive effect on the overall performance in the experiments. This could be due to the fact that the relations taken from UMLS and DrugBank are already implicitly present in the textual data or that some important structured relations are missing or incomplete, e.g., the *has target*-relation from UMLS contains only very few examples. One minor problem is also the way negative examples were constructed from positive examples which does not assure that they really are negative examples for that relation. However, all of these problems can be solved in future work by introducing relations from many other biomedical databases and using existing co-reference resolution implementations.

A major problem when using only dependency paths from natural language text is that context is not considered as a whole. Assertions made in scientific publications do usually hold only under the specified circumstances, which is not reflected by this approach. Furthermore, simple modifications of the dependency paths, like adjectives or adverbs, were also neglected during this study. For example, the second and third feature of Table 4 mention some level being measured, but what is missing is the information about the quality of the level, e.g., high or low. A solution to this problem would be to simply include attributes of nouns and verbs into the dependency paths. This would, however, in some cases also lead to including unnecessary information and moreover result in a much larger number of distinct dependency paths. In future work, ways to circumvent this problem should be investigated. One idea would be to keep the dependency paths as they occur and use textual qualitative attributes to weigh the occurring dependency paths. For example, a triple with a negated dependency path could receive a negative weight and another triple with a dependency path with an attribute “high” should receive a higher weight than one with an attribute “low”. The problem of this approach is that qualities cannot be measured in numbers and they are highly context specific. Moreover, there are more aspects that contribute to the understanding of a sentence which are reflected by other kinds of attributes or constructions, e.g., locational or temporal aspects. This means that a sentence or a statement as a whole is multidimensional with respect to all possible aspects that need to be considered which is impossible to be grasped by solely using dependency paths between entities.

On the other hand, similar problems arise for the use of quantitative data. Including quantitative data from experiments in the form of text, tables or databases is very challenging but the information contained in this data can be very powerful and future work should therefore also investigate towards this direction.

In the experiments LDA was chosen for encoding relation labels. Tests were also done using gPCA, but it did not perform as well as LDA. However, in future work other algorithms can be tried which might learn an even better semantic representation of relation labels than LDA.

Despite the aforementioned problems, the presented results are very promising and they suggest that the use of indirect knowledge can indeed be very helpful in applications dealing with knowledge discovery. Even though the utilized framework does not take local context information into account when extracting information from text, some important correlations between co-occurring pieces of information can be learned from the global context of a concept, where global context of a concept refers to the notion of all connections of this concept to other concepts in the utilized knowledge sources.

## Conclusions

In this study a novel approach for relation discovery in the biomedical domain has been introduced. The approach is based on the combination of information extracted from structured and unstructured data, represented by a graph. The constructed graph allows for the easy integration of heterogeneous information and discovery of indirect connections between biomedical concepts. Given a biomedical relation and example pairs, graph paths are used to create feature vectors with which characteristic path patterns for this relation are learned. For the experimental evaluation of the approach two common biomedical relations; *has target* and *may treat* were used. The results are promising, primarily because they show the feasibility of discovering relations using indirect connections between concepts. In addition, they indicate that the suggested approach can discover the tested relations with an *AUC* of up to 0.8. Furthermore, the application of the approach in these two datasets suggests that it can be applied even when the data is sparse.

The experimental analysis also showed some limitations of the approach. First, there is the problem of incomplete knowledge in the biomedical domain. For example, the extraction of information from text does not take co-references into account. The same problem holds for the structured data sources, where the UMLS is missing some important relations and the existing relations do not cover all currently known facts. Second, the erroneous annotation of the MEDLINE text with MetaMap, e.g., in the case of gene annotation. Finally, the approach does not currently consider the wider context of a statement extracted from a text. However, some important correlations between co-occurring pieces of information can be learned from the global context of entities, which constitutes one of the greatest advantages of the current approach. Towards future work, the focus should lie on addressing the aforementioned problems, by enriching the dependency paths with quantitative and qualitative information extracted from respective attributes that appear in the sentences together with the dependency paths.

## Endnotes

^a^Extracted from MEDLINE.

^b^Archived release statistics of UMLS at http://www.nlm.nih.gov/research/umls/archive/archive_home.html.

^c^synonyms of a concept *c* are all concepts in the metathesaurus connected to *c* by one of the following relations: same_as, clinically_similar, has_tradename, has_alias, gene_encodes_gene_product, mapped_from, SY, RL.

## Additional file

Additional file 1
**Positive and negative examples used for the learning of the “has target” and “may treat” relations.** The examples used for the validation of the methods in the case of drug repositioning are also given. In all cases, UMLS concept identifiers are used.

## References

[1] Swanson DR (1986). Fish oil, raynaud’s syndrome, and undiscovered public knowledge. Perspect Bio Med.

[2] Heim P, Hellmann S, Lehmann J, Lohmann S, Stegemann T. Relfinder: revealing relationships in rdf knowledge bases. Berlin/Heidelberg: Springer: 2009. p. 182–7.

[3] Hoffmann R, Valencia A (2004). A gene network for navigating the literature. Nat Genet.

[4] Frijters R, van Vugt M, Smeets R, van Schaik RC, de Vlieg J, Alkema W (2010). Literature mining for the discovery of hidden connections between drugs, genes and diseases. PLoS Comput Biol.

[5] Cohen T, Schvaneveldt R, Widdows D (2010). Reflective random indexing and indirect inference: a scalable method for discovery of implicit connections. J Biomed Inform.

[6] Srinivasan P, Libbus B, Sehgal AK (2004). Mining medline: Postulating a beneficial role for curcumin longa in retinal diseases. Workshop BioLINK, linking biological literature, ontologies and databases at HLT NAACL.

[7] Hristovski D, Peterlin B, Mitchell JA, Humphrey SM (2005). Using literature-based discovery to identify disease candidate genes. Int J Med Inform.

[8] Vidal ME, Rivera JC, Ibáñez LD, Raschid L, Palma G, Rodriguez H (2014). An authority-flow based ranking approach to discover potential novel associations between linked data. Semantic Web.

[9] Goertzel B, Goertzel IF, Pinto H, Ross M, Heljakka A, Pennachin C (2006). Using dependency parsing and probabilistic inference to extract relationships between genes, proteins and malignancies implicit among multiple biomedical research abstracts. Proceedings of the workshop on linking natural language processing and biology: towards deeper biological literature analysis. BioNLP ‘06.

[10] Lao N, Subramanya A, Pereira F, Cohen WW (2012). Reading the web with learned syntactic-semantic inference rules. Proceedings of the 2012 joint conference on empirical methods in natural language processing and computational natural language learning. EMNLP-CoNLL ‘12.

[11] Knox C, Law V, Jewison T, Liu P, Ly S, Frolkis A (2011). Drugbank 3.0: a comprehensive resource for ‘omics’ research on drugs. Nucleic Acids Res.

[12] Aronson AR. Effective mapping of biomedical text to the UMLS Metathesaurus: the MetaMap program. In: Proceedings/AMIA Annual Symposium. AMIA Symposium: 2001. p. 17–21. http://knowledge.amia.org/amia-55142-a2001a-1.597057/t-001-1.599654/f-001-1.599655/a-003-1.600128/a-004-1.600125. PMC2243666 11825149

[13] Nivre J (2005). Dependency grammar and dependency parsing. MSI Rep.

[14] Dependency Grammar. http://en.wikipedia.org/wiki/Dependency_grammar.

[15] Snow R, Jurafsky D, Ng AY. Learning syntactic patterns for automatic hypernym discovery. In: Advances in Neural Information Processing Systems (NIPS 2004): 2004. p. 1297–304. This is a draft version from the NIPS preproceedings; the final version will be published by April 2005. http://papers.nips.cc/paper/2659-learning-syntactic-patterns-for-automatic-hypernym-discovery.

[16] Bunescu RC, Mooney RJ (2005). A shortest path dependency kernel for relation extraction. Proceedings of the Conference on Human Language Technology and Empirical Methods in Natural Language Processing. HLT ‘05.

[17] Suchanek FM, Ifrim G, Weikum G (2006). Combining linguistic and statistical analysis to extract relations from web documents. Proceedings of the 12th ACM SIGKDD international conference on knowledge discovery and data mining. KDD ‘06.

[18] Liu H, Hunter L, Kešelj V, Verspoor K (2013). Approximate subgraph matching-based literature mining for biomedical events and relations. PloS One.

[19] Yao L, Riedel S, McCallum A (2012). Probabilistic databases of universal schema. Proceedings of the joint workshop on automatic knowledge base construction and web-scale knowledge extraction. AKBC-WEKEX ‘12.

[20] Deerwester S, Dumais ST, Furnas GW, Landauer TK, Harshman R (1990). Indexing by latent semantic analysis. J Am Soc Inf Sci.

[21] Collins M, Dasgupta S, Schapire RE (2001). A generalization of principal components analysis to the exponential family. Advances in neural information processing systems 14 [Neural Information Processing Systems: Natural and Synthetic, NIPS 2001, December 3-8, 2001, Vancouver, British Columbia, Canada].

[22] Blei DM, Ng AY, Jordan MI (2003). Latent dirichlet allocation. J Mach Learn Res.

[23] Yao L, Haghighi A, Riedel S, McCallum A (2011). Structured relation discovery using generative models. Proceedings of the Conference on Empirical Methods in Natural Language Processing. EMNLP ‘11.

[24] Pohl I (1969). Bi-directional and Heuristic Search in Path Problems vol. 104.

[25] Craven M, Kumlien J (1999). Constructing biological knowledge bases by extracting information from text sources. ISMB, vol.1999.

[26] Ravikumar K, Liu H, Cohn JD, Wall ME, Verspoor K (2012). Literature mining of protein-residue associations with graph rules learned through distant supervision. J Biomed Semantics.

[27] Mimno DM, Hoffman MD, Blei DM (2012). Sparse stochastic inference for latent dirichlet allocation. Proceedings of the 29th International Conference on Machine Learning, ICML 2012, Edinburgh, Scotland, UK, June 26 - July 1, 2012.

[28] ClearNLP: Fast and robust NLP components implemented in Java. https://github.com/clearnlp/clearnlp.

[29] TITAN: Distributed Graph Database. https://github.com/thinkaurelius/titan/.

[30] McCallum A, Schultz K, Singh S (2009). FACTORIE: Probabilistic programming via imperatively defined factor graphs. Neural Information Processing Systems (NIPS).

